# Ultraviolet C lamps for disinfection of surfaces potentially contaminated with SARS-CoV-2 in critical hospital settings: examples of their use and some practical advice

**DOI:** 10.1186/s12879-021-06310-5

**Published:** 2021-06-22

**Authors:** Manuela Lualdi, Adalberto Cavalleri, Andrea Bianco, Mara Biasin, Claudia Cavatorta, Mario Clerici, Paola Galli, Giovanni Pareschi, Emanuele Pignoli

**Affiliations:** 1 Medical Physics Unit, Fondazione IRCCS Istituto Nazionale dei Tumori, Via Venezian 1 - 20133, Milan, Italy; 2 Department of Research, Fondazione IRCCS Istituto Nazionale dei Tumori, Milan, Italy; 3 Brera Astronomical Observatory, Italian National Institute for Astrophysics (INAF), Milan/Merate, Italy; 4 Department of Biomedical and Clinical Sciences L. Sacco, University of Milano, Milan, Italy; 5 Don C. Gnocchi Foundation, IRCCS Foundation, Milan, Italy; 6 Department of Pathophysiology and Transplantation, University of Milano, Milan, Italy

**Keywords:** SARS-CoV-2, Environmental contamination, UV-C disinfection, UV-C dosimeters, UV-C lamps

## Abstract

**Background:**

UltraViolet-C (UV-C) lamps may be used to supplement current hospital cleaning and disinfection of surfaces contaminated by SARS-CoV-2. Our aim is to provide some practical indications for the correct use of UV-C lamps.

**Methods:**

We studied three UV-C lamps, measuring their spatial irradiance and emission over time. We quantify the error that is committed by calculating the irradiation time based exclusively on the technical data of the lamps or by making direct irradiance measurements. Finally, we tested specific dosimeters for UV-C.

**Results:**

Our results show that the spatial emission of UV-C lamps is strongly dependent on the power of the lamps and on the design of their reflectors. Only by optimizing the positioning and calculating the exposure time correctly, is it possible to dispense the dose necessary to obtain SARS-CoV-2 inactivation. In the absence of suitable equipment for measuring irradiance, the calculated irradiation time can be underestimated. We therefore consider it precautionary to increase the calculated times by at least 20%.

**Conclusion:**

To use UV-C lamps effectively, it is necessary to follow a few simple precepts when choosing, positioning and verifying the lamps. In the absence of instruments dedicated to direct verification of irradiance, photochromic UV-C dosimeters may represent a useful tool for easily verifying that a proper UV-C dose has been delivered.

## Background

As the Covid-19 pandemic progressed, it became clear that hospitals can be significant epicenters of human to human transmission of the Severe Acute Respiratory Syndrome CoronaVirus 2 (SARS-CoV-2) for healthcare workers, patients and visitors alike. The spread of SARS-Cov-2 is thought mostly to be via the transmission of respiratory droplets and aerosol particles coming from infected individuals [1, 2], and this has led to the conclusion that social distancing and the use of face masks are the most effective tools for containing the transmission of the virus [3]. There is, however, evidence that SARS-CoV-2 can also be transmitted via contaminated surfaces when people touch these surfaces (and then subsequently touch their mouths or eyes), or when the virus on these surfaces becomes airborne again and is then inhaled [1, 4–6]. It should be noted that the use of gloves depends on local regulations and that it is strictly recommended only when cleaning or caring for someone who is sick [1]; presently, patients and visitors do not usually wear gloves in hospital settings.

A recent paper on contamination status of the Zhongnan Hospital of Wuhan University has confirmed that many surfaces in various patient care areas were contaminated with SARS-CoV-2 and that the virus was present on commonly used objects (such as hand sanitizer dispensers, desk surfaces and computer keyboards, coffee dispenser buttons, etc), and on medical equipment (such as pulse oximetry finger clips, oxygen masks, Computed Tomography (CT) scanner and personal protection equipment) [7]. Chin et al. have recently investigated the stability of SARS-CoV-2 in different environmental conditions and on different surfaces [8]. They found that SARS-CoV-2 is relatively stable on smooth surfaces: the virus remained viable up to 1 day on cloth and wood, up to 2 days on glass, 4 days on stainless steel and plastic, and up to 7 days on the outer layer of medical face masks; compared to this, the virus was not present on printing and tissue paper after only 3-h of having been contaminated. Like other coronaviruses, the SARS-CoV-2 virus is characterized by a fragile outer lipid envelope that makes it susceptible to standard disinfection methods. To date, the disinfection of hospital surfaces is carried out using products containing 0.1% chlorine (1000 ppm) or 70–90% ethanol and which are applied following a thorough cleaning with soap and water [9]. Whilst this should in theory be sufficient to eliminate the virus from surfaces, evidences widely reported in the literature [10–12] seem to indicate that the cleaning and disinfection carried out in hospitals may sometimes be suboptimal, resulting in residual contamination. Factors that contribute to the failure to fully sterilize surfaces include: the element of human error inherent in the manual cleaning process, the high turnover of cleaning staff (especially in the case of the outsourcing of cleaning services), incorrect disinfectant contact times, and the incorrect dilution of disinfectant solutions [10–12]. There is, therefore, an urgent need for an effective environmental disinfection strategy that can exist alongside (and additional to) these manual cleaning processes, a strategy that can be non-manual and therefore less susceptible to human error. Further, if this non-manual disinfection process were to take place before the manual cleaning process, this could lead to a safer working environment for the staff, and thus could also lead to cost savings (because the staff would be entering an already decontaminated area).

UltraViolet-C (UV-C) radiation (100-280 nm) has been extensively used for many years for its germicidal effects [13]. Recently, numerous studies have been published on the possible application of UV-C in disinfecting contaminated surfaces by inducing photodimerization in the genomes of SARS-CoV-2 [4, 14–17]. We have used this research to explore the possibility of developing a protocol for the disinfection of potentially contaminated SARS-CoV-2 surfaces through UV-C radiation in our healthcare setting, which is an oncological hospital in Milan without an Accident and Emergency department. To do this, we did the following: we selected a number of potentially contaminated environments; we analyzed two commercially available UV-C lamps as well as a UV-C lamp specially assembled for the study; we built specific supports onto which the lamps could be attached; finally, we established the necessary exposure times to achieve disinfection, basing our calculations both with the use of a spectroradiometer to measure irradiance, and also in the absence of any suitable measuring instrument (i.e. using the manufacturer’s stated measurements). In our opinion, the practical indications for the correct use of UV-C lamps (and in particular for the calculation of the exposure time) are scarcely reported in the literature. To calculate the exposure time correctly, direct irradiance measurements should be performed on the surfaces to be disinfected; however, these measures require tools and skills that are not always available in hospitals. We therefore tried to quantify the error that is committed by calculating the irradiation time based exclusively on the technical data of the lamps (that is, in the absence of technical skills and dedicated tools) or by making direct irradiance measurements. Finally, we tested specific dosimeters for UV-C, easily usable even by non-technical personnel, which can be used both to optimize the positioning of the lamps and to verify that the dose required for sterilization is actually delivered.

Direct verification of SARS-CoV-2 inactivation was not one of the objectives of our work. This verification requires skills and tools present in few hospital settings. Our aim was to provide practical advice so that even non-technical personnel devoid of dedicated tools can use the UV-C lamps correctly starting from the results of the SARS-CoV-2 inactivation tests reported in the literature.

## Methods

Our protocol for the use of UV-C lamps is structured as follows: choice of the environment or the surface to be disinfected; choice and characterization of the lamps to be used; positioning of the lamps; definition of the UV-C dose; calculation of the exposure time in pre-selected reference positions (i.e. in positions where significant contamination is expected) and verification of the delivered UV-C dose in test positions (i.e. where there is doubt about the exposure to the full dose of irradiation that is, in partially shaded positions).

### Choice of environments

There are many environments inside a healthcare facility that can potentially become contaminated with SARS-CoV-2 with various degrees of probability. Environments that have a high probability of being contaminated include the cubicles used for testing patients for SARS-CoV-2, rooms dedicated to Covid patients, and Covid specialized intensive care units. X-ray, CT and visiting rooms have a medium probability of contamination, whereas areas with a low probability of contamination include non-Covid departments and administrative offices. In each of these environments, there are specific surfaces and objects that have a higher probability of being contaminated and thus require utmost care during the disinfection process.

At the outbreak of the Covid pandemic in February 2020, three specific areas in our Institute were set up, which consisted of a) three cubicles for nasopharyngeal swab testing on patients, b) a walk-in clinic for the Covid testing on healthcare professionals working at the hospital, and c) a dedicated inpatient clinic occupying one floor of the hospital for the exclusive treatment of Covid patients. We selected two out of all the potential environments that required a specific disinfection protocol for SARS-CoV-2 to test our disinfection procedure: a cubicle for diagnostic tests (2.0 m width, 2.3 m length, 2.3 m height) and the waiting room for COVID-19 triage (5.5 m width, 10.0 m length, 2.3 m height). We also included in the study a surface with a high probability of contagion: the bed of the CT room (0.7 m width, 2.5 m length).

### Selection and characterization of the UV-C lamps

Numerous studies are presently underway to evaluate the virucidal effects on SARS-CoV-2 of different wavelengths in the UV-C band [4, 14–17]. Considering how inexpensive the mercury lamps are and how easy they are to be procured and to use, we decided to focus our attention on performing tests with ozone-free low pressure mercury lamps producing 254 nm radiation. We tested two commercially available lights, the Sterilight-S72-UV-C (Arenaluci, CastelGoffredo MN, Italy) and the AirZing™ PRO5040 (OSRAM China Lighting Ltd), as well as the Deluxe110, a prototype lamp custom-built specifically for our study by ILT Italy s.r.l. (Albano Laziale Rome, Italy).

Table 1 shows the technical features of the three lamps used as transmitted by the manufacturer; the uncertainties on the nominal wavelength or on the nominal irradiance are not known.

**Table 1:** Technical features of the three UV-C lamps used in this study

Model	Deluxe110	Sterilight-S72-UV-C	AirZing™ PRO5040
Nominal wavelength [nm]	253.7	253.7	253.7
Nominal power [W]	2 × 55	2 × 36	1 × 36
Dimension [mm]	600x240x170	540x210x170	1363x54x78
Weight [Kg]	6.5	4.5	1.3
Nominal irradiance @1 m [μW/cm^2^]	600	220	140
Life time [h]	9000	5000	9000
Rear reflector	Yes	Yes	No
Side reflector	Yes^a^	No	No

^a^Two side reflectors parallel to the luminous elements

The first task was the measurement of the emission stability over time. An irradiance measurement was carried out for each lamp at a distance of 1 m from the geometric center of the luminous elements, with measurements taken at the following times after the lamps were switched on: 15 s, 30 s, 60 s, 120 s, 300 s, 600 s and 900 s. We subsequently mapped the spatial irradiance of the three lamps and their reflectors by performing irradiance measurements in different positions by locating a spectroradiometer on the orthogonal axis of the luminous elements at the following distances from the centre: 1 m, 1.5 m, 2 m, 2.5 m and 3 m. We then repeated the irradiance measurements, positioning the instrument on axes inclined at 45° and 60° with respect to the orthogonal axis, at 0.5 m intervals between 1 m and 3 m. The measurements were repeated both in the plane containing the major axis of the luminous elements and in the plane orthogonal to it. All irradiance measurements were performed 120 s after the lamps were turned on.

The spectral properties of the three lamps and their irradiance were measured using an Ocean Optics HR2000+ spectroradiometer (Ocean Optics Inc., Dunedin, USA), calibrated with reference to a deuterium–halogen source (Ocean Optics Inc. Winter Park, Winter Park, Florida) and in compliance with National Institute of Standards and Technology (NIST) practices recommended in NIST Handbook 150-2E, Technical guide for Optical Radiation Measurements. The detector of our spectroradiometer is a high-sensitivity 2048-element Charge-Coupled Device (CCD) array from Sony. The spectral range is 200–1100 nm with a 25 μm wide entrance slit and an optical resolution of 1.4 nm (Full Width at Half Maximum, FWHM). The cosine-corrected irradiance probe, model CC-3-UV-T, is attached to the tip of a 1 m long optical fibre and is coupled to the spectroradiometer [14].

### Positioning of the UV-C lamps

The optimal positioning of the UV-C lamps and the decision as to whether they should be fixed on the ceiling, the walls, or on a mobile unit, depends on the size and shape of the room to be disinfected, but above all depends on the location of the objects in the room that have a high probability of being contaminated with the virus. The shape of the lamp and the type of reflector are also important factors in achieving a successful outcome. Although fixed lamps are easier to use for the staff, the use of mobile units for the lamps can optimize the irradiation geometry and has the advantage of being adaptable to different environments (and therefore can be more cost efficient).

To evaluate the impact of the choice of the lamps and their positioning on the disinfection process we proceeded as follows. First, we evaluated how the irradiation of the cubicles for nasopharyngeal swabs varies by placing two AirZing™ PRO5040 lamps on the ceiling or on a mobile unit. Irradiance measurements were carried out with the spectroradiometer on the surfaces of greatest interest including the emergency trolley, the medical chair and the headrest of the bed (if present in place of the medical chair). We then evaluated how the irradiation of the waiting room for COVID-19 triage varies by placing two Deluxe 110 lamps on the ceiling compared to two Sterilight-S72-UV-C lamps or two AirZing™ PRO5040 lamps in the same positions. We also evaluated how the irradiation changed by placing two Deluxe 110 lamps on two opposite walls with respect to the scenario with the lamps positioned on the ceiling. Irradiance measurements were carried out on the armrests and on the seats of the chairs. Finally, we evaluated how the irradiation of the bed of the CT room (the item with the higher probability of contagion in the room) varies using the mobile unit equipped with the three lamps enrolled in the study, one at a time. Each lamp was oriented at an angle of about 20° with respect to the longitudinal axis of the bed and at about 80 cm from it, to irradiate both the bed and the control panel on the gantry of the CT. Irradiance measurements were carried out on the control panel and on the side and the headrest of the bed.

**Fig. 1:**
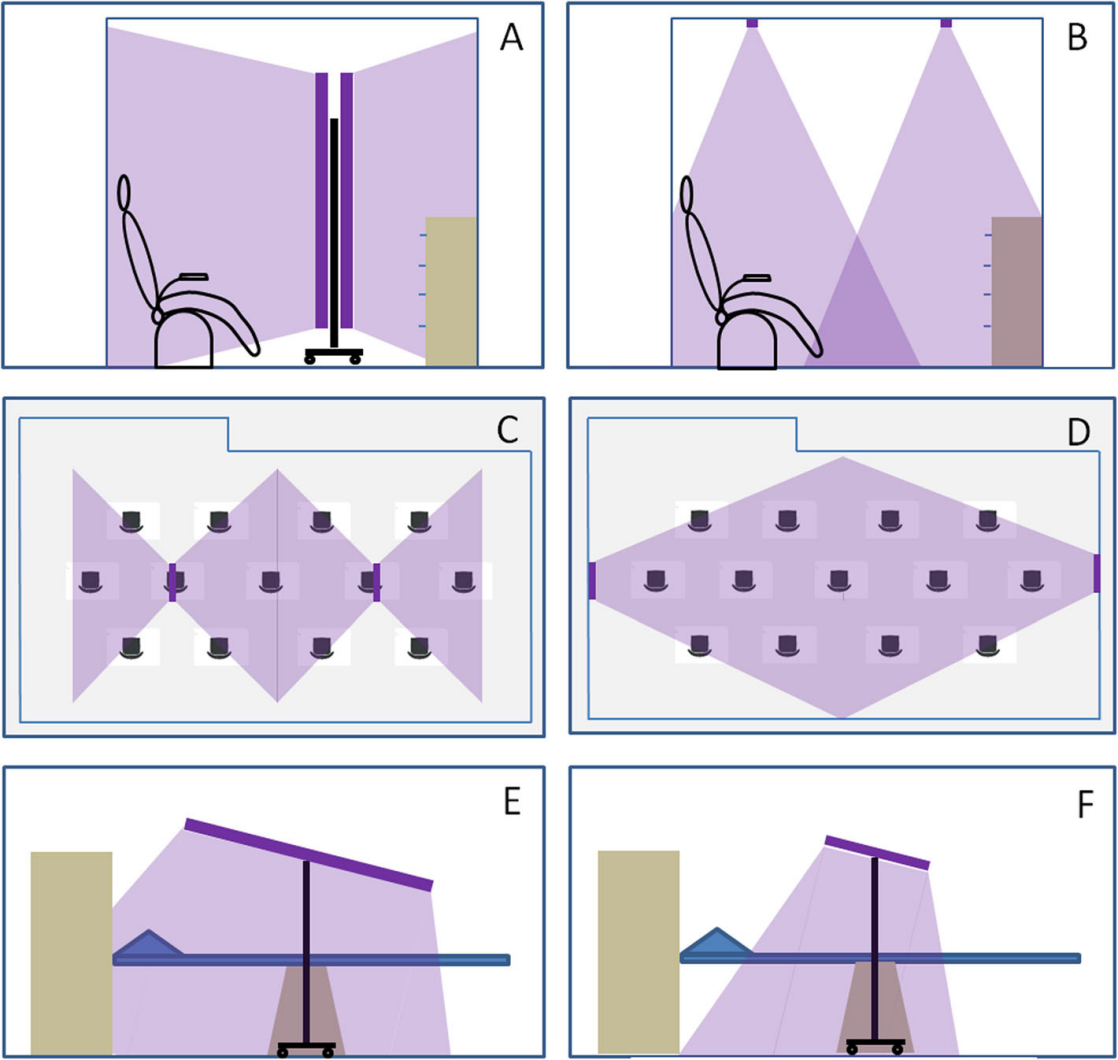
Experimental set up for the evaluation of the impact of the choice of the lamps and their positioning. **A** and **B** show the scheme of the UV-C irradiation obtained in the cubicle for swabs with two AirZing™ PRO5040 lamps mounted on the mobile unit or on the ceiling. **C** and **D** show the scheme of the UV-C irradiation obtained in the waiting room for COVID-19 triage with two Deluxe 110 lamps mounted on the ceiling or on two opposite walls. **E** and **F** show the scheme of the UV-C irradiation of the CT bed obtained with the mobile unit equipped with AirZing™ PRO5040 lamp or with Sterilight-S72-UV-C lamp. In the simplify representation of UV-C radiation only the direct component emerging from the luminous elements was considered

Figure 1 shows the scheme of the scenarios studied to evaluate the impact of the choice and positioning of the lamps on the effectiveness of the disinfection process though UV-C radiation.

### Definition of the disinfection UV-C dose

In our study we planned to deliver a UV-C dose of 3.7 mJ/cm^2^ at 254 nm. This value correspond to the median of the dose values necessary to obtain the inactivation of SARS-CoV-2 resulting from the most recent experiments published on the subject [16]. Since dry biofilms (in which the virus is more resistant) may be present on the surface to be sterilized [11, 18], we believe it is advisable to multiply the dose verified under laboratory conditions by a factor of 10. So, the reference inactivation dose used in the following is 37 mJ/cm^2^.

### Calculation of the exposure time and verification of the delivered UV-C dose

In pre-selected reference positions, we calculated the exposure times using two different approaches: the first approach consisted of calculating the expected irradiance values using the nominal irradiance value provided by the manufacturer corrected by the inverse square law of distance; the corresponding exposure times were obtained by dividing the reference inactivation dose (37 mJ/cm^2^) by the expected irradiance values. For the second approach, we directly measured irradiance over the 250–255 nm wavelength range in the same reference positions; the corresponding exposure times were then corrected to take into account the time needed for the lamp radiance to become fully operational. Finally, the differences between the exposure times calculated with and without direct measurements of irradiance were computed.

To verify that the dose required for sterilization was actually delivered on all exposed surfaces, semi-quantitative measurements of dosage were performed by using disposable UV-C indicators (UVC 100 by Intellego Technologies AB, Gothenburg, Sweden). The UVC 100 dosimeter features a layer of photoactive ink that reacts by changing color according to the amount of UV-C dosage it has been exposed to. The ink can be calibrated to show different tones according to the different dosages. UVC 100-TRI dosimeters are designed to visually indicate an accumulated dose of UV-C of 25, 50and 100 mJ/cm^2^. The photosensitive area changes color as the dosage received increases from yellow (the initial color) to light orange, to dark orange or to dark pink if the dose of 25 mJ/cm^2^, 50 mJ/cm^2^ or 100 mJ/cm^2^ is reached). UVC 100-TRI dosimeters were positioned both in the reference positions (where a dose of 37 mJ/cm^2^ was expected) and where there was doubt about the exposure to the full dose of irradiation (test positions). In details, in the case of the chairs used in the waiting room for COVID-19 triage, the UVC 100-TRI dosimeters were placed both on the armrests of the investigated chairs (reference positions) and on the lateral edge of their seat (test positions). In the case of the cubicles for nasopharyngeal swabs, the dosimeters were placed on the armrest of the medical chair or on the side edge of the bed, if present (reference position) and on the lateral edge of the seat of the medical chair (test position). In case of the CT bed, the UVC 100-TRI dosimeters were placed both on the side edge of the bed and on the headrest (reference position) and on the lateral edge of the headrest (test position). To verify the reliability of the UVC 100-TRI dosimeters, direct irradiance measurements were carried out in all test positions with the previously described spectroradiometer; the corresponding dose values were obtained by multiplying each irradiance value by the length of time of exposure; the comparison of the resulting dose values with the color of the photosensitive area of the dosimeters was finally carried out.

## Results

### Characterization of the UVC-lamps

**Fig. 2:**
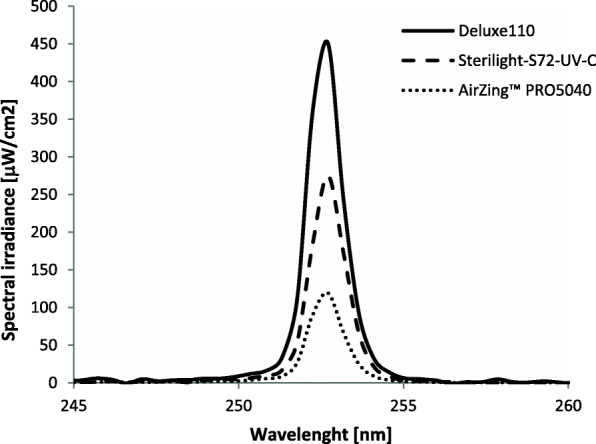
Spectral irradiance at 1 m of the three lamps evaluated in the study. The measurements were made 2 min after switching on

Figure 2 shows the spectral irradiance at 1 m of the three lamps evaluated in the study. As expected, the emission is a function of both the power of the tubes and the characteristics of the reflectors. The integrated irradiance for the Deluxe110, the Sterilight-S72-UV-C and the AirZing™ PRO5040 over the 250–255 nm wavelength range was, respectively, 645 ± 38, 383 ± 37 and 168 ± 12 μW/cm^2^. The comparison of these values with those declared by the manufacturers shows a reasonably good agreement in the case of the AirZing™ PRO5040 (+ 16.7%) and the Deluxe110 (+ 7%). On the contrary, in the case of Sterilight-S72-UV-C, the measured value is + 42% greater than the declared value. This difference could be due to the fact that the value declared by the manufacturer is actually an average value at different angles from the orthogonal axis or an average value over the lifetime of the lamp.

**Fig. 3:**
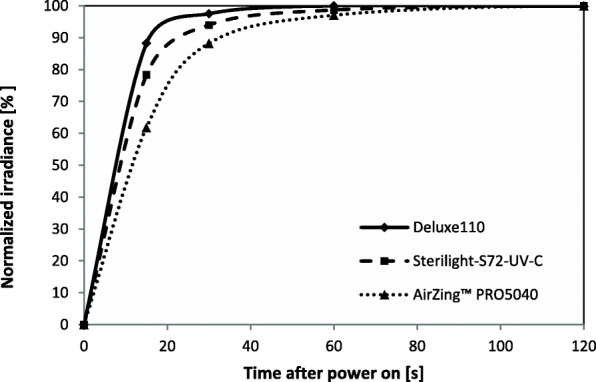
Temporal variation of the normalized integrated irradiance of the three lamps in the first 2 min after being switching on

Figure 3 reports the temporal variation of the emission of the three lamps in the first 2 min after being switching on. To facilitate the comparison, the emissions have been normalized to the maximum. The graph shows that the emission of the Deluxe110 lamp was equal to 88 and 98% of the maximum after 15 s and 30 s respectively. Compared to this, the emission of the AirZing™ lamp was only 62% after 15 s, while after 30 s it reached 88%. The Sterilight-S72-UV-C lamp had an intermediate behavior: its emission was equal to 78 and 95% of the maximum after 15 s and 30 s respectively. For all three lamps, the emission was stable within a variance of 3% after 120 s (and continued to be stable until the fifteenth minute, the duration of the test).

**Fig. 4:**
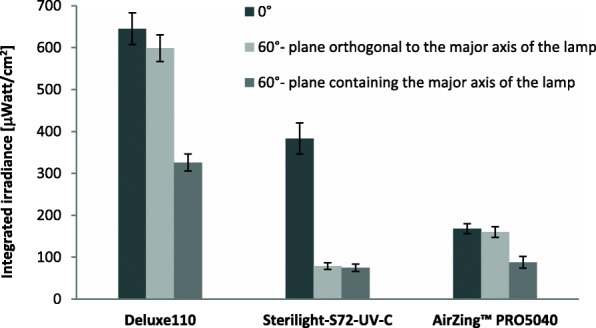
Integrated irradiance (mean and standard deviation of five measurements) of the three lamps at 1 m distance from the center of the luminous elements in three different positions: on the axis orthogonal to the luminous elements, on an axis inclined at 60° to the orthogonal axis in the plane orthogonal to the major axis of the luminous elements and on an axis inclined at 60° to the orthogonal axis in the plane containing the major axis of the luminous elements

Figure 4 shows the integrated irradiance over the 250–255 nm wavelength range of the three lamps at 1 m distance from the center of the luminous elements in three different positions: on the axis orthogonal to the luminous elements, on an axis inclined at 60° to the orthogonal axis in the plane orthogonal to the major axis of the luminous elements and on an axis inclined at 60° to the orthogonal axis in the plane containing the major axis of the luminous elements. The spatial uniformity of the irradiance around each lamp strongly depends on the shape of the lamp itself and on the presence of rear and side reflectors. For example, in the case of the Sterilight-S72-UV-C lamp the absence of side reflectors and the position of the luminous elements (laterally shielded by the lamp structure) greatly limit the uniformity of spatial irradiance. As for the AirZing™ lamp, our results indicate that the spatial irradiance is quite uniform; this is due to the positioning of the luminous element, only rear shielded by the lamp structure. For this lamp, however, the absence of rear and side reflectors does not allow to recover the backscattered irradiance, limiting the useful power in the front part of the lamp.

Finally, the irradiance measurements carried out at different distances from the center of the luminous elements on the orthogonal axis, confirm the decrease in the emission approximately in accordance with the inverse square law of the distance. The deviation from this law increases the more the shape of the lamp differs from that of an ideal point source; the deviation also depends on the presence and geometry of side reflectors. The greatest deviation between the measured value and the expected one, equal to 19%, was recorded in the case of the AirZing™ PRO5040 lamp at a distance of 3 m from the luminous element.

### Positioning of the UV-C lamps

We designed an aluminum support on wheels, equipped with telescopic rods and joints that would enable one or two UV-C lamps to be positioned at the necessary height and with the optimal inclination. The support was equipped with hooks for all the three lamps tested; in the case of the AirZing™ PRO5040, it is also possible to attach two lamps back to back to achieve a 360° exposure. Finally, the power supply of the lamps was connected to a safety circuit equipped with an alarm that sounds when emission begins, along with a timer to set the duration of the emission necessary for disinfection, with a delay function to allow the operator to leave the room in time.

The UV-C irradiation of the cubicle was realized both positioning two AirZing™ PRO5040 lamps on the ceiling and using the mobile unit equipped with two back to back AirZing™ PRO5040 lamps placed vertically. In the first case the results of the irradiance measurements (data not shown) suggest that some surfaces such as the handles of the emergency trolley and the side edge of medical chair were only minimally irradiated while in the case of lamps on the mobile unit all the surfaces of interest were appropriately reached by the UV-C radiation. In detail, with this set up all the measurements confirm the achievement of an irradiance greater than 70 μW/cm^2^ anywhere; the irradiation of the cubicle was therefore carried out efficaciously in less than 10 min.

The results of the irradiance measurements carried out on the chairs of the waiting room with three pairs of the lamps enrolled in the study, confirm that when positioning the lamps on the ceiling, regardless of the power of the lamps and the geometry of the rear reflectors, some potentially contaminated surfaces may not be reached by the UV-C radiation. The best irradiation condition to disinfect the waiting room for COVID-19 triage was obtained by fixing two Deluxe110 lamps on two opposite walls. In this scenario, the exposure time is longer than in the case of ceiling lamps (276 s versus 199 s on a reference chair with two Deluxe 110 lamps) but all surfaces of interest can be efficaciously disinfected. The results reported in Table 2 also confirm that, as aspected, the power of UV-C lamps significantly affects the time required to obtain the target dose.

**Table 2:** Irradiation times calculated with and without direct irradiance measurements

Position	Distance from the lamp^**a**^	Angle^**b**^	Expected irradiance from nominal data	Irradiation time corresponding to expected irradiance	Measured irradiance^**c**^	Irradiation time corresponding to measured irradiance	Time required to compensate for the emission not immediately fully operational	Total irradiation time corresponding to measured irradiance	Difference between measured and expected time
	[m]	[°]	[μWatt/cm^2^]	[s]	[μWatt/cm^2^]	[s]	[s]	[s]	
**CT bed - Mobile support equipped with an AirZing™ PRO5040 lamp**									
Side edge of the bed	0.90	20	165	224	160	231	19	250	10.4%
Headrest	1.40	45	71	521	46	804	17	821	36.6%
**Cubicle for nasopharyngeal swab - Mobile support equipped with two AirZing™ PRO5040 lamps**									
Medical chair	0.85	20	199	187	191	194	17	211	11.4%
Side edge of the bed	1.30	30	83	446	74	500	18	518	13.9%
**Waiting room for COVID-19 triage - Two Deluxe110 lamps on the ceiling**^**d**^									
First chair	1.90	0	166	223	196	189	10	199	−12.1%
Second chair	2.60	45	89	416	89	416	9	425	2.1%
**Waiting room for COVID-19 triage - Two AirZing™ PRO5040 lamps on the ceiling**^**d**^									
First chair	1.90	0	39	949	42	881	18	899	−5.6%
Second chair	2.60	45	21	1762	13	2846	17	2863	38.5%
**Waiting room for COVID-19 triage - Two Sterilight-S72-UV-C lamps on the ceiling**^**d**^									
First chair	1.90	0	61	607	109	339	13	352	−72.4%
Second chair	2.60	45	33	1121	30	1233	13	1246	10.0%
**Waiting room for COVID-19 triage - Two Deluxe110 lamps on the walls**^**d**^									
First chair	2.30	0	113	327	139	266	10	276	−18.5%
Second chair	4.60	45	28	1321	28	1321	9	1330	0.7%

^a^The distance is calculated from the reference position to the center of the luminous elements

^b^Angle between the axis orthogonal to the luminous elements and the line joining the reference position and the center of the luminous elements

^c^Irradiance measurements were made two minutes after switching on

^d^Irradiance measurements were made by switching on only one lamp at a time

To disinfect the bed of the CT room, we used the mobile unit equipped with a UV-C lamp to irradiate both the bed and the control panel on the gantry of the CT. The irradiance measurements carried out with the three lamps enrolled in the study confirm that lamps with long luminous elements should be preferred for the disinfection of elongated surfaces. In fact, only in the case of AirZing™ PRO5040 lamp, our measurements indicated an irradiance greater than 40 μW/cm^2^ anywhere so that the UV-C irradiation of the CT bed can be done efficaciously in about 10 min, therefore also between one patient and the next. In the case of Sterilight-S72-UV-C and Deluxe110 lamps, the shorter length of the luminous elements determines a significant decrease in irradiance in the plane of the luminous elements themselves; as a result, some positions such as the control panel and the ends of the bed were not reached by the UV-C radiation making disinfection ineffective.

### The calculation of the exposure time and the verification of the delivered UV-C dose

Table 2 shows the comparison between the irradiation times calculated with and without direct irradiance measurements in twelve reference positions (i.e. in positions where significant contamination is expected and where it was established to deliver the reference inactivation dose). The fourth column shows the irradiance values calculated by using the irradiance at 1 m, supplied by the manufacturer, corrected by the inverse square law of distance between each reference position and the source. The corresponding irradiation times, reported in the fifth column, were obtained by dividing the reference inactivation dose (37 mJ/cm^2^) by the expected irradiance values. The uncertainty on the calculation of the irradiation times is less than 1% as the only source of error is the measurement of the distance between the lamp and the reference position. The sixth column reports the irradiance values measured at the reference positions; the corresponding irradiation times were reported in the seventh column. The uncertainty on the calculation of the irradiation time is, in this case, within 3%, which corresponds to the uncertainty of measurement of our spectroradiometer. The eighth column reports the time required to compensate for the emission to be fully operational and, finally, the ninth column reports the total irradiation times corresponding to the measured irradiance values.

In 9 out of 12 reference positions, the agreement between the irradiation times calculated with and without direct irradiance measurements is within 20%. In some cases, however, this agreement was achieved as a combination of two larger errors in opposite directions. For example, concerning the second reference position of the Sterilight-S72-UV-C, the overestimation of the irradiance that would be made in the absence of direct measurements at angles greater than 45° was partially compensated by the fact that the actual irradiance is, in the case of this lamp, much greater than the nominal. The latter is also the cause of the difference between the irradiation times calculated with and without direct irradiance measurements for the first reference position of the Sterilight-S72-UV-C (72.4%). In the two remaining reference positions (second reference position of the CT bed scenario and second reference position of the waiting room scenario with AirZing™ PRO5040 lamps), the differences between measured and expected time (36.6 and 38.5%) were due to the not-frontal location of the reference positions with respect to the lamps: in the absence of side reflector, the irradiance at angles greater than 45° with respect to the orthogonal axis of the luminous elements may be critically lower than that declared by the manufacturer.

Finally, as can be deduced from the results reported in Table 2, if the time required to compensate for the emission to be fully operational is ignored, the error on the irradiation time is about 10% for exposures of a few minutes or negligible for exposures longer than 10 min.

Table 3 shows the results obtained by irradiating the UVC 100-TRI dosimeters placed in 12 reference positions (where a dose equal to 37 mJ/cm^2^ was expected) and in 10 test positions (where a lower dose was expected since, at same length of time of exposure, the irradiance was lower due to greater distance and deflating inclination). In all the reference positions and in 7 test positions the color of the sensitive area of the dosimeter after UVC irradiation was intermediate between light orange and dark orange or similar to dark orange, suggesting that the target dose of 37 mJ/cm^2^ was probably achieved. Finally, in 3 test positions the color of the sensitive area was hardly distinguishable from light orange, indicating that a dose of just over 25 mJ /cm^2^ has been reached.

**Table 3:** Comparison between the dose calculated from direct measurements of irradiance and the dose indicated by the UVC-100 TRI photocromic dosimeters

Position	Measured irradiance [μWatt/cm^2^]	Expected dose [mJ/cm^2^]	UVC-100 TRI color^**a**^	Is the target dose of 37 mJ/cm^**2**^ reached?
**CT bed - Mobile support equipped with an AirZing™ PRO5040 lamp**				
Reference position: side edge of the bed	160	37	LO-DO	Probably yes
Reference position: headrest	46	37	LO-DO	Probably yes
Test position: lateral edge of the headrest	44	35	LO-DO	Probably yes
**Cubicle for swab - Mobile support equipped with two AirZing™ PRO5040 lamps**				
Reference position: medical chair (armrest of the chair)	191	37	LO-DO	Probably yes
Reference position: side edge of the bed	74	37	LO-DO	Probably yes
Test position: medical chair (lateral edge of the seat)	69	35	LO-DO	Probably yes
**Waiting room for COVID-19 triage - Two Deluxe110 lamps on the ceiling**				
Reference position: first chair (armrest of the chair)	196	37	DO	Yes
Test position: first chair (lateral edge of the seat)	190	36	DO	Yes
Reference position: second chair (armrest of the chair)	89	37	LO-DO	Yes
Test position: second chair (lateral edge of the seat)	84	35	LO-DO	Probably yes
**Waiting room for COVID-19 triage - Two AirZing™ PRO5040 lamps on the ceiling**				
Reference position: first chair (armrest of the chair)	42	37	LO-DO	Probably yes
Test position: first chair (lateral edge of the seat)	39	34	LO-DO	Probably yes
Reference position: second chair (armrest of the chair)	13	37	LO-DO	Probably yes
Test position: second chair (lateral edge of the seat)	9	26	LO	Probably no
**Waiting room for COVID-19 triage - Two Sterilight-S72-UV-C lamps on the ceiling**				
Reference position: first chair (armrest of the chair)	109	37	LO-DO	Probably yes
Test position: first chair (lateral edge of the seat)	100	34	LO-DO	Probably yes
Reference position: second chair (armrest of the chair)	30	37	LO-DO	Probably yes
Test position: second chair (lateral edge of the seat)	23	28	LO	Probably no
**Waiting room for COVID-19 triage - Two Deluxe110 lamps on the walls**				
Reference position: first chair (armrest of the chair)	139	37	LO-DO	Probably yes
Test position: first chair (lateral edge of the seat)	130	35	LO-DO	Probably yes
Reference position: second chair (armrest of the chair)	28	37	LO-DO	Probably yes
Test position: second chair (lateral edge of the seat)	23	30	LO	Probably no

^a^*LO-DO* intermediate between light orange and dark orange, *LO* very similar to light orange, *DO* very similar to dark orange

## Discussion

Healthcare infections are infections that patients and healthcare workers contract while in a health care setting. They can be caused by a range of microorganisms including bacteria, fungi and viruses present in the hospital environment and can result in serious illness including, since the beginning of 2020, COVID-19. Decontamination of environmental surfaces is critical in reducing and preventing the transmission of pathogens. Thus, in healthcare facilities appropriate cleaning and disinfection protocols need to be carefully selected with particular attention given to surfaces with a high probability of contagion. Given the limitations of manual disinfection methods, it is extremely important to introduce optimized automated non-touch disinfection methods, such as hydrogen peroxide vapor and UV-C irradiation. Automated non-touch methods avoid human error during the disinfection procedure, allows for frequently repeat disinfection cycles on high-touch surfaces (for example the CT bed and the chair needed to conduct nasopharyngeal swabs that need to be disinfected between one patient and the next), and to limit the risk of contaminating cleaning staff who perform manual disinfection after the automated cleaning has taken place.

In the ultraviolet spectrum, UV-C has the highest disinfection capacity with an efficacy peak at 265 nm [13]. Presently, the radiation wavelength most used is that supplied by low pressure mercury lamps (254 nm), which have the advantage of being cheap and easily available. The main drawback on their use is that there are strict environmental protocols on the disposal of mercury [19]. The 270-280 nm radiation produced by LED is as effective as the 254 nm radiation [20]. LED technology has the advantage that lamps can be produced with very small dimensions and customizable geometries and they don’t contain harmful metals, although their main disadvantage is that they are very expensive to fabricate. Recently, the use of the 222 nm radiation lamp has been promulgated with the idea that this shorter wavelength radiation might be less harmful to those exposed [15]. However, these lamps are still very expensive and very bulky, and they still require careful supervision to avoid damage to the skin and to the eyes. Numerous studies are underway to establish the disinfection power of the UV-B (280-320 nm) and UV-A (320-400 nm) bands. At the time of writing, there were very few published results [17] that indicate the effectiveness of these spectral bands to disinfect SARS-CoV-2 contaminated surfaces.

To date, only mercury lamps can therefore be used to disinfect surfaces in hospital environments. There are numerous mercury lamps available on the market, varying in power, geometry and the shape of the reflectors. To achieve the necessary disinfection, however, it is essential to choose the correct lamps for the environment they are going to be used in, and above all to position them in an optimal way, something that requires both suitable equipment and the adequate training of staff to be able to use this equipment; these conditions are often not present in healthcare facilities.

The intention of our study, therefore, was to present practical and simple advice for UV-C disinfection which includes guidance as to the choice and positioning of the lamps, a method to reduce the error on the calculation of the exposure time and provide a way to check irradiation by using proper UV-C dosimeters. The choice of the lamp depends on the type of environment or surface to be disinfected; in the case of smaller rooms or elongated surfaces (such as beds), low power lamps (32 or 36 W) with long tubes (120 or 150 cm) are to be preferred. In the case of large rooms, it is necessary to use more powerful (and thus more expensive) lamps (36 × 2 W or 55 × 2 W). In both cases, the presence of side and rear reflectors made of highly reflective material allows to significantly increase the spatial uniformity of the irradiance and to recover the radiation delivered in non-useful direction.

The positioning of the lamps also depends on the type of environment or surface to be disinfected: lamps on mobile supports seem generally better at disinfecting surfaces, whereas lamps fixed on ceilings or walls are preferable when whole rooms need to be disinfected. In choosing the number of lamps to be installed, attention must be paid to surfaces at angles greater than 45° with respect to the irradiation source; in this case, especially in the absence of side and rear reflectors, it is advisable to increase the number of lamps used. It is also important to remember that only surfaces that are exposed to the direct light from the lamp will be irradiated; the reflected component of the primary radiation from common materials contributes minimally to sterilization. This is certainly the main limitation of the UVC disinfection method which must therefore be proposed in addition to and not as a substitute for standard decontamination procedures.

It is the calculation of the exposure time for successful disinfection that poses the greatest challenge to establishing a disinfection protocol. The most recent studies on the subject provide the dose value necessary to obtain the inactivation of SARS-CoV-2. However, if the virus is present in dry biofilms deposited on surfaces, then the resistance to sterilization could be greater. Pending more studies on this subject, it is prudent to multiply the dose obtained in the laboratory by a factor of 10 in real-life environments. Once the disinfection dose is known, the irradiation time can be calculated by dividing this value by the irradiance of the lamp, obtained by applying the inverse square law to the irradiance value at 1 m supplied by the manufacturer. In the absence of direct measurements of the irradiance, there are numerous reasons for error that can affect the irradiation time calculated, the most significant of which seem to be the position of the object at angles greater than 45° with respect to the irradiation source (in the absence of proper side reflectors) and not compensating for the time required for the emission to be fully operational. In the case of lamps whose shape is very different from that of an ideal point source, also the application of the inverse square law of the distance can lead to a not negligible underestimation of the irradiation time. It is therefore considered precautionary to increase the calculated times by at least 20%. Our results suggest that even with this correction, significant underestimation of the irradiation time cannot be excluded; one method to easily verify that a proper UV-C dose has been delivered is to position photochromic UV-C dosimeters on any surfaces where there is doubt about the exposure. The color reached by the sensitive area of the dosimeter will confirm the achievement of the target dose or will suggest increasing the exposure time or changing the inclination of the lamps.

The UV-C disinfection protocol must include a safety section that takes into account the consequences of UV overexposure on the skin and eyes of any operators present. Therefore, the use of the UV-C lamps and the possible presence of operators must be planned in accordance with the most recent guidelines on the matter [21, 22]. When using open-air UVC systems such as those described in the study, a few seconds at a distance of a couple of meters or less from the lamps may be enough to exceed the recommended exposure limits [21]. During the lighting of the lamps, the operators must therefore not be present in the room undergoing disinfection. To ensure this, it is necessary to define a safety protocol that requires, for example, to arrange an interlock on the door connected to the lamp power supply, or to purchase lamps equipped with a presence sensor. It is also recommended that on / off switches for UVC lamps not be located in the same location as general room lightning; instead, they should be located or password protected to ensure that they are not accidentally turned on. The only unavoidable exposure is that of UV lamps commissioning workers who can perform a direct measurement of irradiance using a hand-held radiometer. In this case, workers are required to use Personal Protective Equipment (PPE) which consists of eyewear and facial mask compliant to ISO 4007:2018 [23] and clothing and gloves known to be nontransparent to UVC penetration. Regarding the possible damage induced by UV-C on materials, it must be remembered that most of the organic based materials can become damaged if they are not properly protected to UV rays. This damage, called UV degradation, affects many natural and synthetic polymers including some rubbers, neoprene and polyvinyl chloride (PVC). With too much exposure, these materials can fade in color, lose strength, become less flexible and finally crack. Certain inks and dyes can be affected as well. This problem, called photodegradation or phototendering, causes objects like textiles, artwork and polymers to: change color, fade in color and produce a chalky surface. Not all materials become damaged by UV radiation. Many silicones are generally UV-stable, as well as acrylic and types of glass, etc. At the time of writing, damage to materials in the hospital environments due to UV-C light disinfection was not reported [11]. We have recently initiated a study to evaluate the photodegradation of wooden furnishings and the damage induced on the synthetic leather of the upholstery of the CT beds and of the swab armchairs. To date, the repetition of 700 disinfection cycles (25,900 mJ/cm^2^) did not show any difference compared to the unexposed material. However, the study will be extended up to the delivery of 50,000 mJ/cm^2^.

The main limitation of our study is not to have decontamination measurements on biofilms in which the virus may be embedded. A specific study on the subject has already been activated to verify UV-C induced inactivation on different surfaces.

## Conclusion

Disinfection of surfaces potentially contaminated by SARS-CoV-2 with UV-C lamps can be achieved by following some practical rules: choose UV-C lamps of proper power and shape equipped with side and rear reflectors made of highly reflective material; increase the exposure time calculated using the nominal irradiance by at least 20%; verify that a proper UV-C dose has been delivered by positioning photochromic UV-C dosimeters on any surfaces where there is doubt about the exposure.

## Data Availability

The datasets used and/or analysed during the current study are available from the corresponding author on reasonable request.
